# Remote Sensing Image of The Landsat 8–9 Compressive Sensing via Non-Local Low-Rank Regularization with the Laplace Function

**DOI:** 10.3390/e25030523

**Published:** 2023-03-17

**Authors:** Guibing Li, Weidong Jin, Jiaqing Miao, Ying Tan, Yingling Li, Weixuan Zhang, Liang Li

**Affiliations:** 1 School of Electrical Engineering, Southwest Jiaotong University, Chengdu 611756, China; lgb527@swun.edu.cn (G.L.); liangli@my.swjtu.edu.cn (L.L.); 2 Key Laboratory for Computer Systems of State Ethnic Affairs Commission, Southwest Minzu University, Chengdu 610041, China; ty7499@swun.edu.cn (Y.T.); liyingling@swun.edu.cn (Y.L.); 3 China-ASEAN International Joint Laboratory of Integrated Transportation, Nanning University, Nanning 530299, China; 4 School of Mathematics, Southwest Minzu University, Chengdu 610041, China; jiaqing_miao@swun.edu.cn; 5 School of Computer Science and Engineering, Southwest Minzu University, Chengdu 610041, China; onlychoise@163.com

**Keywords:** compressed sensing (CS), non-local (NL), Laplace function (LF), ADMM, Landsat 8–9 remote sensing images (LRSIs)

## Abstract

Utilizing low-rank prior data in compressed sensing (CS) schemes for Landsat 8–9 remote sensing images (RSIs) has recently received widespread attention. Nevertheless, most CS algorithms focus on the sparsity of an RSI and ignore its low-rank (LR) nature. Therefore, this paper proposes a new CS reconstruction algorithm for Landsat 8–9 remote sensing images based on a non-local optimization framework (NLOF) that is combined with non-convex Laplace functions (NCLF) used for the low-rank approximation (LAA). Since the developed algorithm is based on an approximate low-rank model of the Laplace function, it can adaptively assign different weights to different singular values. Moreover, exploiting the structural sparsity (SS) and low-rank (LR) between the image patches enables the restored image to obtain better CS reconstruction results of Landsat 8–9 RSI than the existing models. For the proposed scheme, first, a CS reconstruction model is proposed using the non-local low-rank regularization (NLLRR) and variational framework. Then, the image patch grouping and Laplace function are used as regularization/penalty terms to constrain the CS reconstruction model. Finally, to effectively solve the rank minimization problem, the alternating direction multiplier method (ADMM) is used to solve the model. Extensive numerical experimental results demonstrate that the non-local variational framework (NLVF) combined with the low-rank approximate regularization (LRAR) method of non-convex Laplace function (NCLF) can obtain better reconstruction results than the more advanced image CS reconstruction algorithms. At the same time, the model preserves the details of Landsat 8–9 RSIs and the boundaries of the transition areas.

## 1. Introduction

The transmission, reception, and storage of Landsat 8–9 remote sensing images (RSIs) is a critical practical challenge in remote sensing (RS). Therefore, the collection time and processing of massive Landsat 8–9 images have become hot research topics. Compressed sensing (CS) aims to sample/compress the original image using part of the image data (or the corresponding frequency-domain data) and then reconstruct the sampled/compressed data at the terminal by obtaining the reconstructed image [1] close to or beyond the quality of the original data. Generally, CS theory exploits the sparsity and low-rank prior information of the original image to compress the RSI through a data compression method and to recover and reconstruct the compressed data when required.

Nevertheless, the Landsat 8–9 RSIs involve huge real matrices, and this huge data volume (the compressed file size of each group of Landsat 8–9 RSIs exceeds 1GB) imposes a long data transmission time [2], while many practical applications require fast remote sensing data acquisition, limiting the practical application of Landsat 8–9 RSIs. For example, fast data acquisition enables timely assessment of the losses caused by natural disasters, and real-time monitoring of ground objects also depends on the rapid transmission and analysis of RSIs. Therefore, CS technology has prominent practical applications in reconstructing Landsat 8–9 RSIs. At the same time, the compression perception model of an RSI poses a great practical application potential in improving the energy efficiency of imaging sensors [3]. Given that the proportion of random measuring signals acquired by CS in the image is relatively low (typically 10–25%), measuring signal transmission [4] has a significant advantage in time and space storage.

The traditional CS model usually employs a regularization model, compresses the original image using the frequency-domain random sampling (FDRS) method, and then obtains the compressed data [5] with sparsity. Consequently, many image CS reconstruction models are built on practical problem situations. For instance, the traditional CS reconstruction method can be effectively solved using the 11-norm of the sampled signal, i.e., the sparsity of the original signal, and the low-rank function replacement (LRFR) method [6], which effectively solves the CS reconstruction problem. In recent years, the concept of sparsity has been developed into various complex forms, including model-based or Bayesian, non-local sparsity, and structural sparse/group sparsity [7]. Thus, CS data can be reconstructed by exploiting the high correlation between the sparse coefficient, while some experimental studies have demonstrated that the non-convex optimization method (NCOM) based on CS has better image reconstruction results. Indeed, NCOM-based reconstructed images are often higher in visual effect and numerical accuracy than the convex optimization method (COM) [8,9]. To exploit the high correlation between the sparse coefficient and non-convex optimization and find a solution algorithm with relatively high computational efficiency, [10] introduced a method for solving the nuclear norm low-rank approximation model (LRAM) using the SVD algorithm, which yields an easily solved convex optimization problem (COP) [10] by minimizing the sum of all singular values in the image. However, each singular value of the optimization problem in image reconstruction has its practical physical meaning, and therefore, each singular value in any real problem should be treated differently [11]. However, all the singular values have been averaged in the nuclear norm LRAM, limiting the algorithm’s ability and flexibility.

Spurred by the findings presented above, we suggest a CS reconstruction model for the Landsat 8–9 RSI based on low-rank approximation (LRA) of non-local associative non-convex Laplace functions (NL-NCLF), which adaptively assign weights to different singular values [12]. Extensive experimental studies demonstrate that the reconstruction results of NCOMs are more accurate than COMs, but the algorithms are slightly complex and impose a greater computational complexity [13]. In the ongoing big data era, overemphasizing the algorithm’s computational time is no longer an important target; therefore, NCOMs have good practical value and prospects. Hence, the core priority is the model to make full use of the prior information of the original signal (such as sparsity and low-rank) in the CS theory of Landsat 8–9 RSIs [14,15].

Generally, the image patches of Landsat 8–9 RSIs present a strong sparsity and have an approximate structural low rank [16]. Therefore, due to the low rank of the structural information in the Landsat 8–9 RSIs, we employ the SVD method to solve the CS model and thus obtain better CS reconstruction results [17]. Moreover, for the image reconstruction, we utilize the regularization model of the low-rank approximate penalty of the non-local combined non-convex Laplace function, which is based on structural self-similarity in the total variation framework [8,9,12]. Our model is a non-convex CS reconstruction model involving a regularization/penalty constraint, low-rank approximation, and image patch grouping [18]. Substituting the low-rank regularization with a non-local and Laplace function enhances the CS reconstruction accuracy compared to common low-rank substitution methods, e.g., nuclear norm, with extensive theoretical analysis proving our algorithm’s effectiveness and stability.

## 2. Background

In the CS theory of Landsat 8–9 RSIs, random linear measurement/sampling is conducted in the corresponding Fourier transform domain (FTD) of the RSIs *x*, and then the sampling data *y* of the Fourier frequency domain (FFD) is obtained. The original Landsat 8–9 RSIs *x* are recovered through image reconstruction with the sampling data *y*. In general, the real Landsat 8–9 RSIs are not absolutely sparse but approximately sparse under a certain transformation domain, i.e., they can be transformed into a compressible signal. The sparsity or compressibility of image signals is an important premise and the theoretical basis for CS. Hence, a remote sensing image *x* can be represented by a linear combination of a set of bases $\Psi =\left\{\eta_{i}\right\},\;i=1,2,\cdots ,n$, where $\eta_{i}\in C^{N}$ are the sparse bases, i.e., x=Ψα. Specifically, *y* can be expressed as y=Φx=ΦΨα, where $x\in C^{N}$, $y\in C^{M}$ and $\Phi \in C^{M\times N}$ is the measurement/sampling matrix, M<N. Since M<N, matrix Φ is not a full rank, i.e., multiple reconstruction results $x\in C^{N}$ can be generated using the same measurement *y*. Generally, if the measurement matrix Φ satisfies the limited isometry property (RIP) condition [12,16], CS theory can guarantee the perfect reconstruction of signal *x* by utilizing the sparse (or compressed) signal *y*. Prior information about the image *x* must be known to reconstruct a unique perfect image *x* from the measured data *y*. The traditional CS method is to recover remote sensing images by using the sparsity of images *x*, and to satisfy y=Φx, the following constraint optimization problem is employed:

(1) $$\begin{array}{c} x=\underset{x}{\arg\min}\;{∥\alpha ∥}_{0}\\ s.t.\;\;\;y=\Phi \Psi \alpha , \end{array}$$

where ${∥\cdot ∥}_{0}$ is a pseudo-norm counting the number of non-zero elements in α. However, minimizing the norm ${∥\cdot ∥}_{0}$ is an NP-hard problem. Therefore, we recover the RSI signal from the random measurement/sampling *y* by using the convex norm $l_{1}$ instead of the non-convex norm $l_{0}$ and then solve the norm $l_{1}$ optimization problem. The optimal model obtained by substituting norm $l_{1}$ for the norm $l_{0}$ is a convex optimization model, and therefore multiple solution algorithms such as an iterative contraction algorithm (ICA) [19], Bregman splitting algorithm (BSA) [20], and alternating direction multiplier method (ADMM) [21,22] can effectively solve this problem. Recent studies have demonstrated that replacing the norm $l_{1}$ with a non-convex norm achieves better CS reconstruction results [16].

By modeling the high correlation between sparse coefficients, the uncertainty of unknown signals can be significantly reduced and afford a more accurate CS reconstruction. The structural sparsity of Landsat 8–9 RSI is particularly important in establishing the CS model for RSIs. Usually, image structure information presents a rich repeatability, so the non-local self-similarity (NLSS) principle of an image structure can be obtained by combining the non-local method [14] and the simultaneous sparse coding (SSC) mechanism [23]. One of the key issues in the CS model is the minimization of the rank function. Because the kernel norm is the minimum convex envelope of the rank function, the rank function in the traditional CS model is often relaxed to the kernel norm (the sum of all singular values of the matrix). However, many works in the literature have proven that using kernel norm to approximate the rank of a matrix has many weaknesses, especially when the matrix has large singular values, the inaccuracy of this approximation is particularly obvious. Therefore, it is particularly necessary to study how to construct a more accurate rank approximation function (RAF) and establish a corresponding low-rank matrix (LRM) recovery model based on it. In recent years, the non-convex function approximation (NNFA) of the LRM’s rank function has widely concerned many scholars. A large number of experimental results have proven that these non-convex rank approximation functions are more accurate than the convex function approximation of the kernel norm. The approximate rank function method of the non-convex function (NCF) can not only avoid the NP-hard problem but also provide the optimal solution. The non-convex function replacement (NCFR) can usually provide more scalable solutions. The comparison between the non-convex Laplace replacement function (NCLRF) and the kernel norm replacement function (KNRF) under standard conditions shows that the NCLRF model can better approximate the rank function than the kernel norm model when solving the rank minimization problem. Therefore, it is very meaningful to explore the non-convex regularized low-rank approximation (NCRLRA) model, establish a fast and effective solution algorithm, and apply it to practical problems such as the compressed sensing of a remote sensing image (RSI). This paper suggests a variational framework for CS reconstruction using non-local structural sparsity (NLSS) and non-convex low-rank approximation (NCLRA) [23].

## 3. Regularized CS Reconstruction Model Based on Non-Local and Non-Convex Approximate Low-Rank Functions

This section presents a new regularized CS reconstruction model with non-local and an NCLRA, comprising a patch grouping to describe the self-similarity of the images and an NCLRA for low-rank enhancement. Our method assumes that the non-local self-similarity in Landsat 8–9 RSIs is very rich. This assumption implies that for each sample image patch ${\hat{x}}_{i}$ ($\sqrt{n}\times \sqrt{n}$) sample patch at position (*i*), a sufficient number of similar image patches can be found by performing a k-nearest neighbor (KNN) search algorithm in a local window, e.g., 100×100, when ${\hat{x}}_{i}\in C^{n}$, namely:

(2) $$G_{i}=\left\{i_{j}|∥{\hat{x}}_{i}-{\hat{x}}_{i,j}∥<T\right\},$$

where *T* is a predefined threshold and $G_{i}$ represents the set of locations corresponding to the similar image patch. After the patch is grouped, we obtain the data matrix $X_{i}=\left[x_{i_{0}},x_{i_{1}},\ldots ,x_{i_{m-1}}\right]$, $X_{i}\in C^{n\times m}$. For each sample image patch $x{}_{i}$, each column of $X{}_{i}$ represents a patch similar to $x{}_{i}$ (including $x{}_{i}$). Since these patches have similar structures, the data matrix $X{}_{i}$ formed is low-rank.

In practical applications, $X{}_{i}$ may be interfered with by noise, thus deviating from the ideal low-rank constraint (LRC). Thus, a better representation of the data matrix $X_{i}$ is $X_{i}=L_{i}+W_{i}$, where $L_{i}$ and $W_{i}$ represent the low-rank matrix (LRM) and the Gaussian noise matrix (GNM), respectively. By solving the optimization problem in Equation (3), the LRM is reconstructed:

(3) $$\begin{array}{c} L_{i}=\underset{L_{i}}{\mathrm{argmin}rank}\;\left(L_{i}\right)\\ s.t.\;\;{∥X_{i}-L_{i}∥}_{F}^{2}\leq \sigma_{w}^{2}, \end{array}$$

where ${∥\cdot ∥}_{F}^{2}$ represents the Frobenius norm and $\sigma_{w}^{2}$ is the variance of additive Gaussian noise. Although the low-rank convex substitution method has good theoretical guarantees, the optimization method of non-convex substitution for the rank minimization problem may obtain better recovery results.

This paper uses a smooth non-convex function (Laplace function) as the alternative to the low-rank function. Nowadays, several works employ the nuclear norm approximate low-rank function, which provides equal weight to all the singular values in the image patch. However, in many practical situations, the singular values have different physical meanings and should be treated differently [11,12], which is particularly prominent for RSIs. For example, larger singular values represent low-frequency information in Landsat 8–9 RSIs, while smaller values represent high-frequency information and noise. The Laplace function $\phi \left(x\right)=1-e^{-x/\varepsilon }$ used in this paper is closer to the pseudo-norm $l_{0}$ than the nuclear norm, and thus the sum of the singular values of the Laplace function is closer to the rank function than the nuclear norm. Additionally, the advantage of the Laplace function is that it automatically assigns different weights to each singular value. Based on the above observations, we propose a non-convex low-rank substitution model of the Laplace function, defined as the norm form in Equation (4):

(4) $${∥X∥}_{\varepsilon }=\phi \left(\sigma \left(X\right)\right)=\sum_{i=1}^{n}1-e^{-\sigma_{i}\left(X\right)/\varepsilon \;},$$

where ε denotes a smaller constant value. Note that function ${∥X∥}_{\varepsilon }$ is the sum of *n* singular value functions of the matrix *X*. So it is smooth and non-convex. Laplace non-convex functions can substitute rank function, which has been proven better when considering information theory [24,25]. On these grounds, the non-convex low-rank approximation optimization model (NC-LRAOM) of Equation (5) is proposed to solve $L_{i}$.

(5) $$\begin{array}{c} L_{i}=\underset{L_{i}}{\arg\min}\;{∥L_{i}∥}_{\varepsilon }\\ s.t.{∥X_{i}-L_{i}∥}_{F}^{2}\leq \sigma_{w}^{2}, \end{array}$$

In practice, the constrained minimization problem can be solved with an unconstrained minimization problem, namely:

(6) $$L_{i}=\underset{L_{i}}{\arg\min}\;{∥X_{i}-L_{i}∥}_{F}^{2}+\lambda {∥L_{i}∥}_{\varepsilon },$$

By selecting the appropriate λ, Equation (6) can be made equivalent to Equation (5). For each sample image patch, an approximate low-rank matrix $L_{i}$ of the matrix $X_{i}$ can be obtained by solving Equation (6), and for each extracted sample image patch the low-rank was enforced on the non-local similar image patch set. Hence, a new non-convex CS reconstruction model is proposed following the proposed low-rank regularization term. The specific CS reconstruction scheme is as follows:

(7) $$\left(\hat{x},{\hat{L}}_{i}\right)=\underset{x,L_{i}}{\arg\min}\;{∥y-\Phi x∥}_{2}^{2}+\eta \sum_{i}\;\left\{{∥{\tilde{R}}_{i}x-L_{i}∥}_{F}^{2}+\lambda {∥L_{i}∥}_{\varepsilon }\right\},$$

where ${\tilde{R}}_{i}x≐\left[R_{i_{0}}x,R_{i_{1}}x,\ldots ,R_{i_{m-1}}x\right]$ is a matrix comprising a group of similar patches of each sample image patch $x_{i}$. The proposed regularized model with non-local combined non-convex approximate low-rank function (NL-NCALF) simultaneously utilizes the group sparsity of similar image patches and the non-convexity of rank minimization, thus being able to obtain better reconstruction results. In the next section, the proposed objective function will be solved effectively using the minimization method of the non-convex function instead of the low-rank function.

## 4. Landsat 8–9 Remote Sensing Image CS Reconstruction Algorithm

The proposed CS reconstructed algorithm of the Landsat 8–9 RSI can be solved by minimizing the objective function and the low-rank matrix (LRM) $L{}_{i}$ of the whole image *x*. For the initial estimate of the unknown image *x*, we first extract the sample image patch $x_{i}$ at each pixel *i* in each direction and assign a similar set to each image patch $x_{i}$, as described in Section 3. Then we solve the following minimization problem for each image patch as follows:

(8) $$L_{i}=\underset{L_{i}}{\arg\min}\;\eta {∥{\tilde{R}}_{i}x-L_{i}∥}_{F}^{2}+\lambda {∥L_{i}∥}_{\varepsilon },$$

Equation (8) is solved using Theorem 1:

**Theorem 1.**  *Given $Z\in R^{m_{1}\times m_{2}}$, the minimum value of Equation (9):*

(9) $$\arg\min_{X}\;{∥X∥}_{\varepsilon }+\frac{\beta }{2}{∥Z-X∥}_{F}^{2},$$

*is given by a weighted singular value threshold, i.e.,:*

(10) $$X=UD_{{}^{\nabla \phi }/{}_{\beta }}V^{H},$$

*where $Z=USV^{H}$ and $D_{{}^{\nabla \phi }/{}_{\beta }}\in R^{m_{1}\times m_{2}}$ is a diagonal matrix, $D_{\frac{\nabla \phi }{\beta }}\left(i,i\right)={\left(S\left(i,i\right)-\frac{\nabla \phi \left(\sigma_{i}\right)}{\beta }\right)}_{+}$, $\nabla \phi \left(\sigma_{i}\right)=\frac{1}{\varepsilon }\exp\left(-\frac{\sigma_{i}}{\varepsilon }\right)$ denotes the gradient of ϕ at position $\sigma_{i}$, and $\sigma_{i}$ is the ith singular value of X.*

**Proof.**  Equation (4) shows that the function ${∥X∥}_{\varepsilon }$ is the sum of *n* singular value functions of matrix *X*, then Equation (9) can be expressed in the form of Equation (11):

(11) $$\arg\min_{X}\;\sum_{i=1}^{m}\phi \left(\sigma_{i}\left(X\right)\right)+\frac{\beta }{2}{∥X-Z∥}_{F}^{2},$$

where $X,\;Z\in C^{m_{1}\times m_{2}}$, and the optimal solution of Equation (11) can be obtained using the general weighted singular value threshold [26,27], namely:

$$X=UD_{{}^{\nabla \phi }/{}_{\beta }}V^{H},$$

where $Z=USV^{H}$, $D_{\frac{\nabla \phi }{\beta }}\left(i,i\right)={\left(S\left(i,i\right)-\frac{\nabla \phi \left(\sigma_{i}\right)}{\beta }\right)}_{+}$, $\nabla \phi \left(\sigma_{i}\right)=\frac{1}{\varepsilon }\exp\left(-\frac{\sigma_{i}}{\varepsilon }\right)$, Consequently, the conclusion of Theorem 1 can be confirmed. □

In the case of a real matrix, the proposed CS reconstruction model for the Landsat 8–9 RSI is non-convex, so it is not expected to find its global minimum. However, the weighted singular threshold algorithm can obtain the minimum value (possibly the local minimum value) of Equation (9). Note that even though the weighted threshold method is only a local minimum, the value of the objective function still decreases. In the experiment, we set $w_{}^{\left(0\right)}={\left[1,1,\ldots ,1\right]}^{T}$.

According to [28], weighting $l_{1}$-norm performs much better than $l_{1}$-norm in approximating $l_{0}$-norm and generally produces better image CS perception reconstruction results. Similarly, the experimental results in the next section demonstrate that the Laplace function produces CS reconstruction results better than the nuclear norm. By solving the following minimization problem to obtain $L_{i}$, we can further reconstruct the whole Landsat 8–9 RSI:

(12) $$x=\underset{x}{\arg\min}\;{∥y-\Phi x∥}_{2}^{2}+\eta \sum_{i}\;{∥{\tilde{R}}_{i}x-L_{i}∥}_{F}^{2},$$

when the measurement matrix Φ is the Fourier transform matrix (the Fourier transform is one of the important transforms in the field of Landsat 8–9 RSI processing), Equation (12) can be quickly solved using the alternating direction multiplier method (ADMM) [29]. In this case, first, the augmented Lagrangian form of Equation (12) can be expressed as in Equation (13):

(13) $$\left(x,z,\mu \right)=\underset{x}{\arg\min}\;{∥y-\Phi x∥}_{2}^{2}+\beta {∥x-z+\frac{\mu }{2\beta }∥}_{2}^{2}+\eta \sum_{i}\;{∥{\tilde{R}}_{i}z-L_{i}∥}_{F}^{2},$$

where $z\in C^{N}$ is an auxiliary variable, $\mu \in C^{N}$ is a Lagrange multiplier, and β is a positive constant. The advantage of ADMM is that Equation (13) can be divided into two subproblems, where both can find their closed solutions. Applying ADMM to Equation (13), the iterative form of the following Equation (14) can be obtained:

(14) $$\begin{array}{c} z^{\left(l+1\right)}=\underset{z}{\arg\min}\;\beta^{\left(l\right)}{∥x^{\left(l\right)}-z+\frac{\mu^{\left(l\right)}}{2\beta^{\left(l\right)}}∥}_{2}^{2}+\eta \sum_{i}\;{∥{\tilde{R}}_{i}z-L_{i}∥}_{F}^{2}\\ x^{\left(l+1\right)}=\underset{x}{\arg\min}\;{∥y-\Phi x∥}_{2}^{2}+\beta^{\left(l\right)}{∥x-z^{\left(l+1\right)}+\frac{\mu^{\left(l\right)}}{2\beta^{\left(l\right)}}∥}_{2}^{2}\\ \mu^{\left(l+1\right)}=\mu^{\left(l\right)}+\beta^{\left(l\right)}x^{\left(l+1\right)}-z^{\left(l+1\right)}\\ \beta^{\left(l+1\right)}=\rho \beta^{\left(l\right)}, \end{array}$$

In Equation (14), ρ>1 is a constant. By determining $x^{\left(l\right)}$, $\mu^{\left(l\right)}$, $\beta^{\left(l\right)}$, and $z^{\left(l+1\right)}$, a closed solution can be obtained:

(15) $$z^{\left(l+1\right)}={\left(\eta \sum_{i}\;{\tilde{R}}_{i}^{T}{\tilde{R}}_{i}+\beta^{\left(l\right)}I\right)}^{-1}\left(\beta^{\left(l\right)}x^{\left(l\right)}+\frac{\mu^{\left(l\right)}}{2}+\eta \sum_{i}\;{\tilde{R}}_{i}^{}L_{i}\right),$$

Note that $\sum_{i}{\tilde{R}}_{i}^{T}{\tilde{R}}_{i}$ is a diagonal matrix. Therefore, Equation (15) can be easily calculated.

The sub-problems of *x* and *y* can be calculated using Equation (16):

(16) $$\left(\Phi^{H}\Phi +\beta^{\left(l\right)}I\right)x=\left(\Phi^{H}y+\beta^{\left(l\right)}z^{\left(l+1\right)}-\frac{\mu^{\left(l\right)}}{2}\right),$$

where Φ is the Fourier transform matrix (FTM), Φ=DF and where *D* and *F* represent the down-sampling matrix and Fourier transform matrix (FTM), respectively. In other words, the sub-problems of *x* and *y* can be solved from the image space to the Fourier frequency domain space by Equation (16). Equation (17) can be obtained by replacing Φ with DF in Equation (16) and executing Fourier transform on both sides of the equation.

(17) $$F\left({\left(DF\right)}^{H}DF+\beta^{\left(l\right)}I\right)F^{H}Fx=F{\left(DF\right)}^{H}y+F\left(\beta^{\left(l\right)}z^{\left(l+1\right)}-\frac{\mu^{\left(l\right)}}{2}\right),$$

So, we simplify Equation (17) and calculate *x* by taking the inverse Fourier transform, namely:

(18) $$x^{\left(l+1\right)}=F^{H}\left\{{\left(D^{T}D+\beta^{\left(l\right)}\right)}^{-1}\left(D^{T}y+F\left(\beta^{\left(l\right)}z^{\left(l+1\right)}-\frac{\mu^{\left(l\right)}}{2}\right)\right)\right\},$$

By updating *x* and *z* according to Equation (14), μ and β can be easily calculated.

After the unknown image *x* is calculated, the low-rank matrix $L_{i}$ is updated using Equation (8), from which we obtain the updated $L_{i}$ that is used to recalculate the estimate of image *x*. This process is repeated until the algorithm meets the convergence condition.

The proposed CS reconstruction model for the Landsat 8–9 RSIs is non-convex. Therefore, the hot start method is used to preprocess the CS reconstruction images. When the reconstruction results reach a certain accuracy, the above ADMM method is used to solve the proposed model and obtain higher accuracy. To save computing time, image patch grouping is not updated after each iteration but at the end of an outer cycle.

## 5. Data Sources

This study employs Landsat 8–9 RSIs for all simulation experiments obtained from the US Geological Survey Center for Earth Resource Observation and Science (EROS). Landsat 8 is the eighth satellite of the US Landsat Missions (Landsat), successfully launched on an Atlas-V rocket from Vandenberg Air Force Base, California, on 11 February 2013, originally known as the Landsat Data Continuity Mission (LDCM). Landsat 8 carries an operational land imager (OLI) and a thermal infrared sensor (TIRS). The OLI measures the spectrum’s visible, near-infrared, and shortwave infrared portions (VNIR, NIR, and SWIR). The TIRS measures the temperature of land surfaces in two thermal bands with a new technology that applies quantum physics to detect heat. The OLI Land Imager includes nine spectral bands with 30-m multi-spectral spatial resolutions, which include a 15-m panchromatic band. This study’s first set of satellite images was the Landsat-8 LITP product of the blue band (30-m) with a wavelength range of 450–515 nm. Landsat 9 is the ninth satellite of the US Landsat Missions (Landsat), successfully launched from the Vandenburg Space Force Base, California, on 27 September 2021. Landsat 9 Carries the second-generation land imager (Operational Land Imager 2, OLI-2) built by Ball Aerospace & Technologies and the second-generation thermal infrared sensor (Thermal Infrared Sensor 2, TIRS-2) built by NASA Goddard Space Flight Center. The OLI–2 captures images of the Earth’s surface in visible, near-infrared, and shortwave-infrared bands, increasing the radiative measurement accuracy from 12 to 14 bits with Landsat 8 and slightly improving the overall signal-to-noise ratio. The OLI-2 land imager includes nine spectral bands with a 30-meter spatial resolution, which includes a 15-meter panchromatic band. The TIRS-2 measures the thermal infrared radiation or heat of the Earth’s surface in two bands that perform better than the thermal band of the Landsat 8. This study’s second set of satellite images is the Landsat 9 L2SP product of the red band (30-meter) with a wavelength range of 640–670 nm. Table 1 summarizes the locations and dates of the observed images.

The Landsat 8 RSIs were ordered in four different geographical regions: Antarctica; Selkirk in Manitoba, Canada; Flathead Lake in Montana, USA; Lincoln in Washington, USA. Antarctica is mainly composed of ice sheets, glaciers, and snow, while Selkirk in Manitoba, Canada, is a grassland area with relatively flat terrain, a Precambrian shield, and its northern end is the permafrost layer. Flathead Lake in Montana, USA, is in the state’s northeast and is known for its rich rocks and plains. It includes rich land features, such as water, mountain, forest, and vegetation. Lincoln has abundant landforms, abundant rainfall, and large desert areas in the east.

The Landsat 9 remote sensing images (RSIs) [30] were ordered in five different geographical regions: Lake Abitibi in Ontario, Canada; Yeosu, South Korea; Shanghai, China; Huangshi, Hubei, China; Erenhot, Inner Mongolia, China. The Lake Abitibi waters range from western Lake Forest to the St. Lawrence River in eastern Cornwall. Lake Abitibi stretches between Ontario and Quebec and includes many land features, such as lakes, forests, cities, and farmland. Yeosu is located in the Yeosu Peninsula, the southernmost tip of the Korean Peninsula, belonging to South Jeolla Province, and it includes the ocean, coastline, and city. Shanghai is located on the west coast of the Pacific Ocean, where the Yangtze River and Huangpu River converge. Its land features include rivers, oceans, cities, islands, and peninsulas. Huangshi City is located on the south bank of the middle reaches of the Yangtze River, facing the Yangtze River in the northeast and Huanggang City across the river, and it is rich in minerals. In the northwest and middle of the city, lakes such as Baoan, Sanshan, and Daye form a plain lake marshland, while the rest are low mountains and hills. Erenhot is flat and gently inclined from southwest to northeast. The land features include forests, deserts, grasslands, villages, and vegetation.

The objective of selecting Landsat 8–9 RSIs of different regions is to ensure that the newly-developed model performs very well for diverse land cover types.

## 6. Experimental Results and Analysis

**Figure 1:**
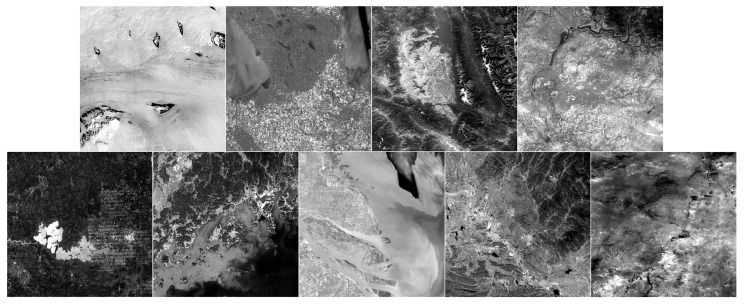
Synthetic Landsat 8–9 RSIs used for model evaluation. The first row presents the synthetic Landsat 8 images (from left to right: the first image was collected in Antarctica on 22 February 2018; the second image was collected in Selkirk, Manitoba, Canada, on 7 October 2022; the third image was collected in Lake Flathead, Montana, USA, on 29 September 2017; the last image was collected in Lincoln, Washington, USA on 15 October 2015). The second row presents the synthetic Landsat 9 RSIs (from left to right: the first image was collected in Lake Abitibi, Ontario, Canada, on 11 October 2022; the second image was collected in Yeosu, Korea, on 12 October 2022; the third image was collected in Shanghai, China on 8 April 2022; the fourth image was collected in Huangshi, Hubei, China on 13 October 2022; the last image was collected in Erenhot, Inner Mongolia, China on 28 July 2022).

**Figure 2:**
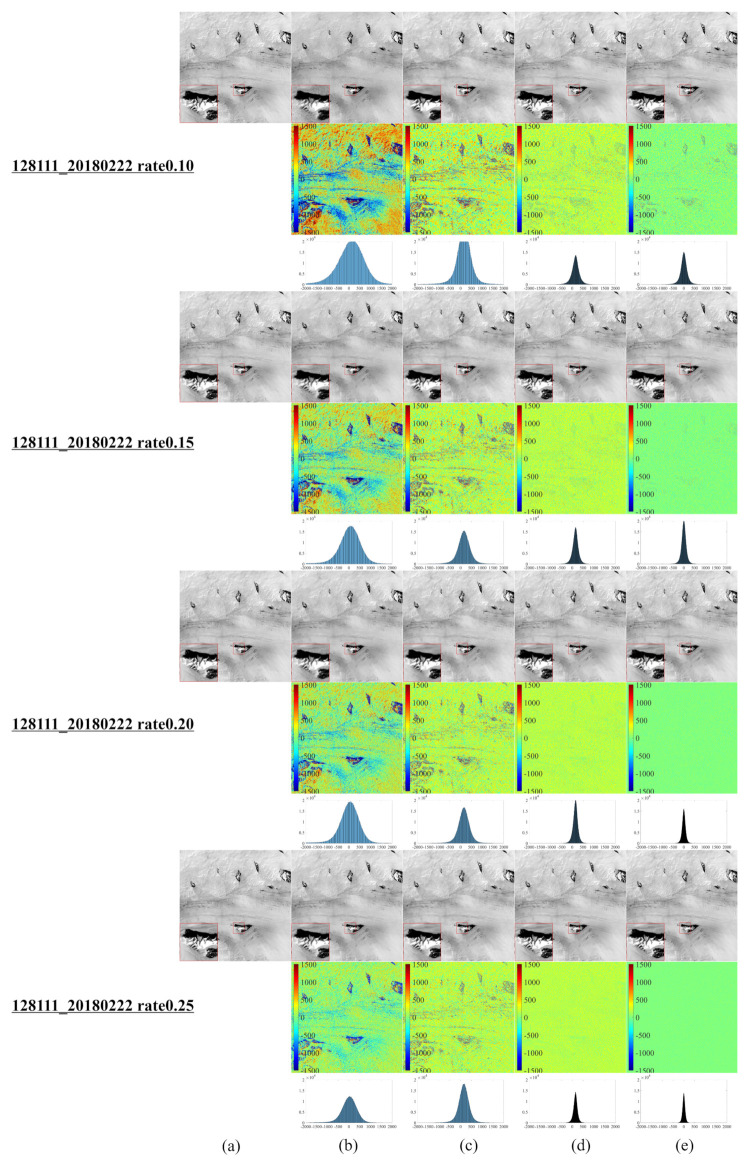
Reconstruction Results using Sampling Rates of 10%, 15%, 20%, and 25% for a Landsat 8 RSI in Antarctica, (**a**) original Landsat 8 RSI of Antarctica, (**b**–**e**) of the first row (10%), the fourth row (15%), the seventh row (20%), and the tenth row (25%) are the reconstruction result images of NL-SRF-CS, KCS-GSR, NLDR-CS, and NL-Laplace-CS models, respectively. The red square area in the lower left corner of each image is the magnified form of the selected area in the image, which is convenient for observing the details of the reconstruction results. The images in the second, fifth, eighth, and eleventh rows are the differences between the reconstructed and original images corresponding to each sampling ratio. The third, sixth, ninth, and twelfth rows show the histograms of the corresponding difference image.

**Figure 3:**
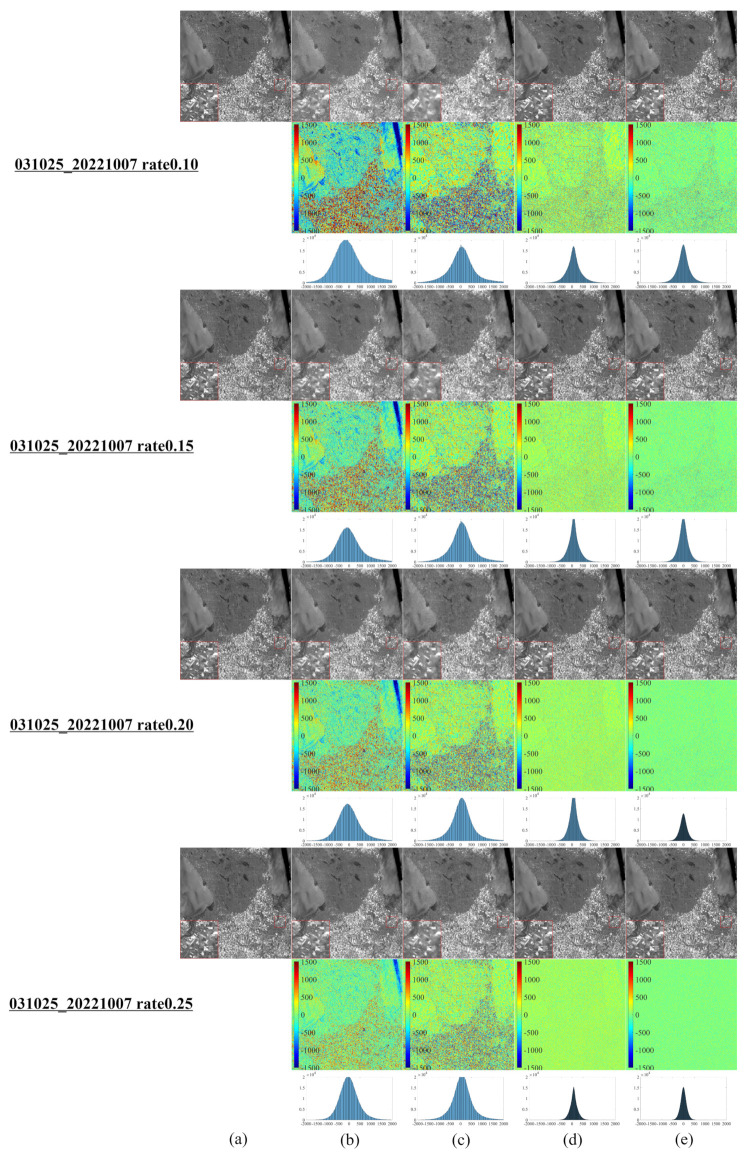
CS reconstruction result images of a Landsat 8 RSI in Selkirk, Manitoba, Canada, are the same as the second image on the left of the top row of Figure 1. (**a**–**e**) The specific details of the images are the ones presented in Figure 2.

**Figure 4:**
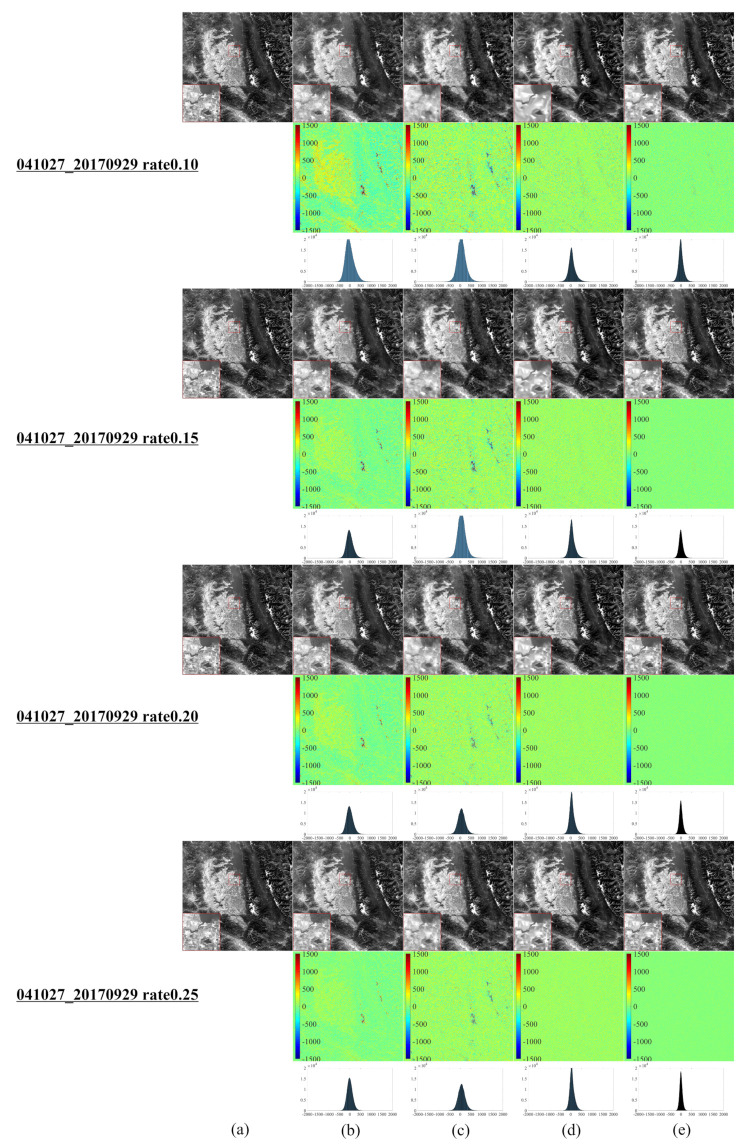
CS reconstruction result images of a Landsat 8 RSI in Lake Flathead, Montana, USA ((**a**–**e**) similar to Figure 2 and the third to the left image in the first line of Figure 1).

**Figure 5:**
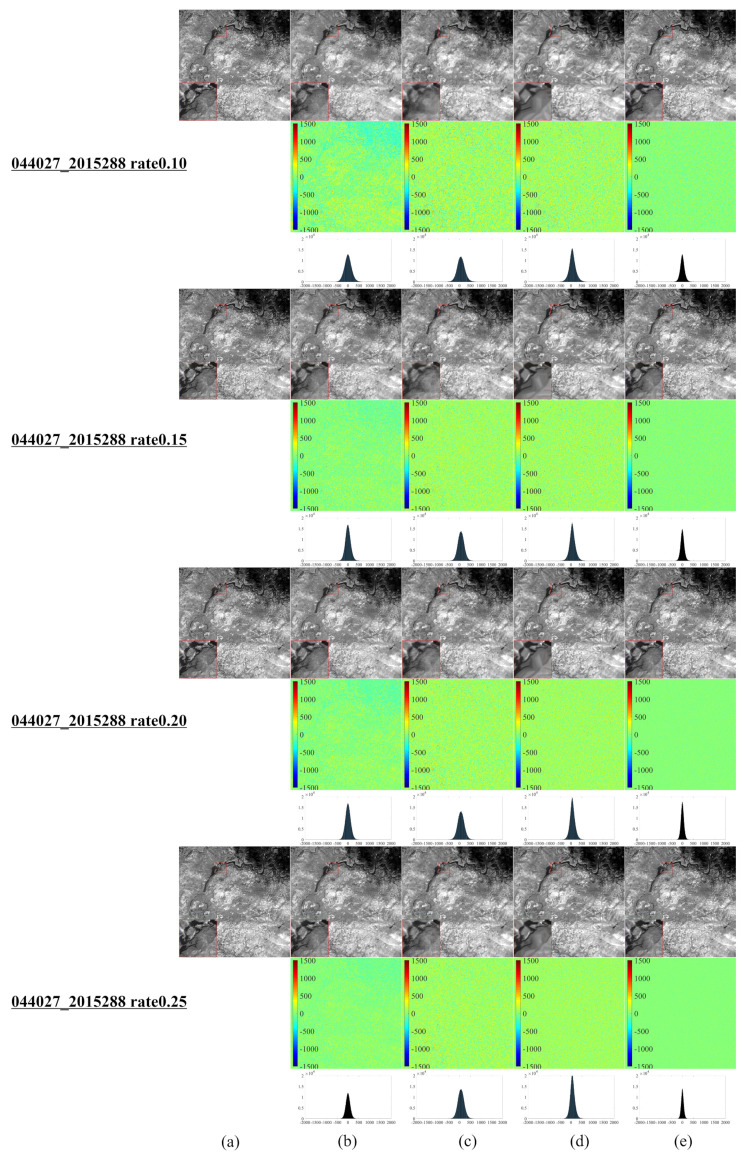
CS reconstruction result images of a Landsat 8 RSI in Lincoln, Washington, USA ((**a**–**e**) similar to Figure 2 and the last image in the first line of Figure 1).

**Figure 6:**
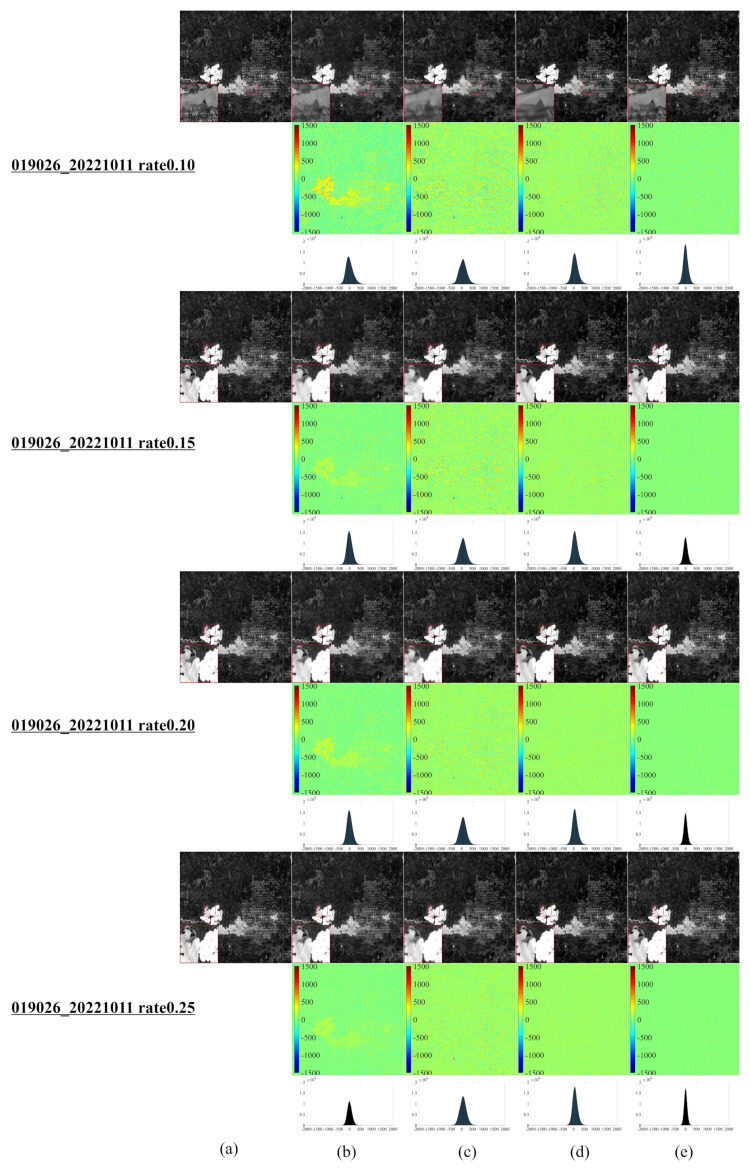
CS reconstruction result images of a Landsat 9 RSI in Lake Abitibi, Ontario, Canada, using various models ((**a**–**e**) similar to Figure 2 and the first image on the left in the second row of Figure 1).

**Figure 7:**
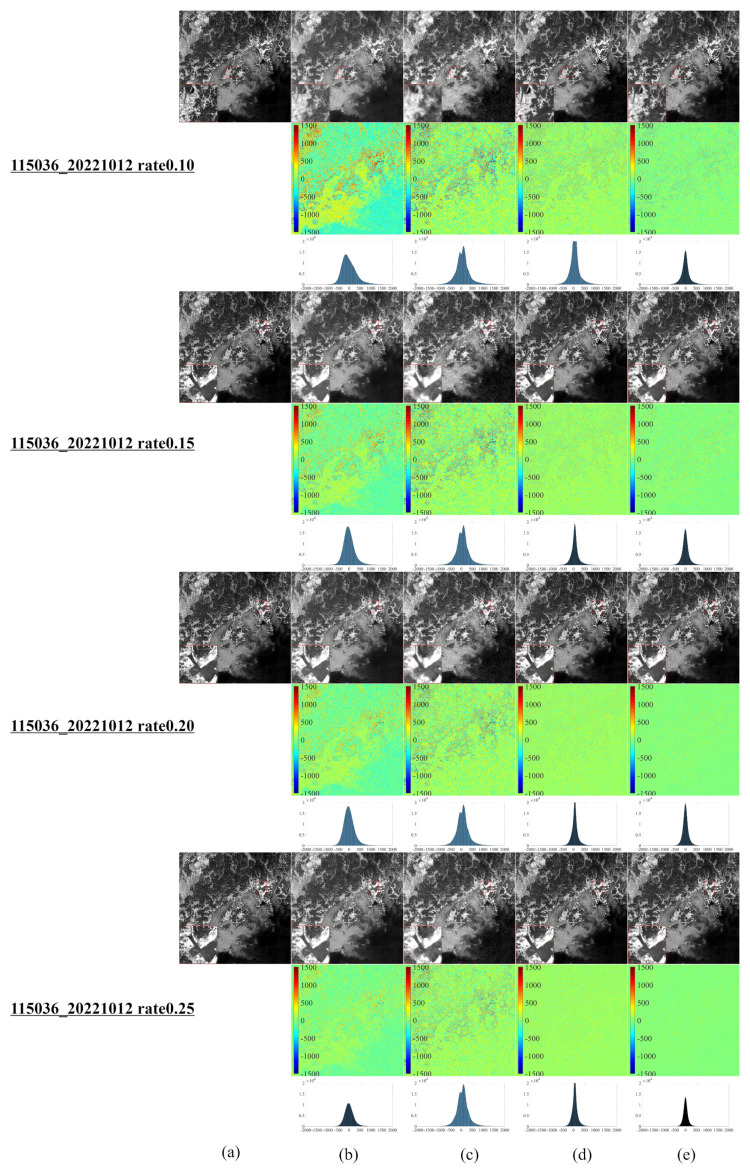
Landsat 9 remote CS reconstruction of Yeosu using various models ((**a**–**e**) similar to Figure 6 and the second left image in the second row of Figure 1).

**Figure 8:**
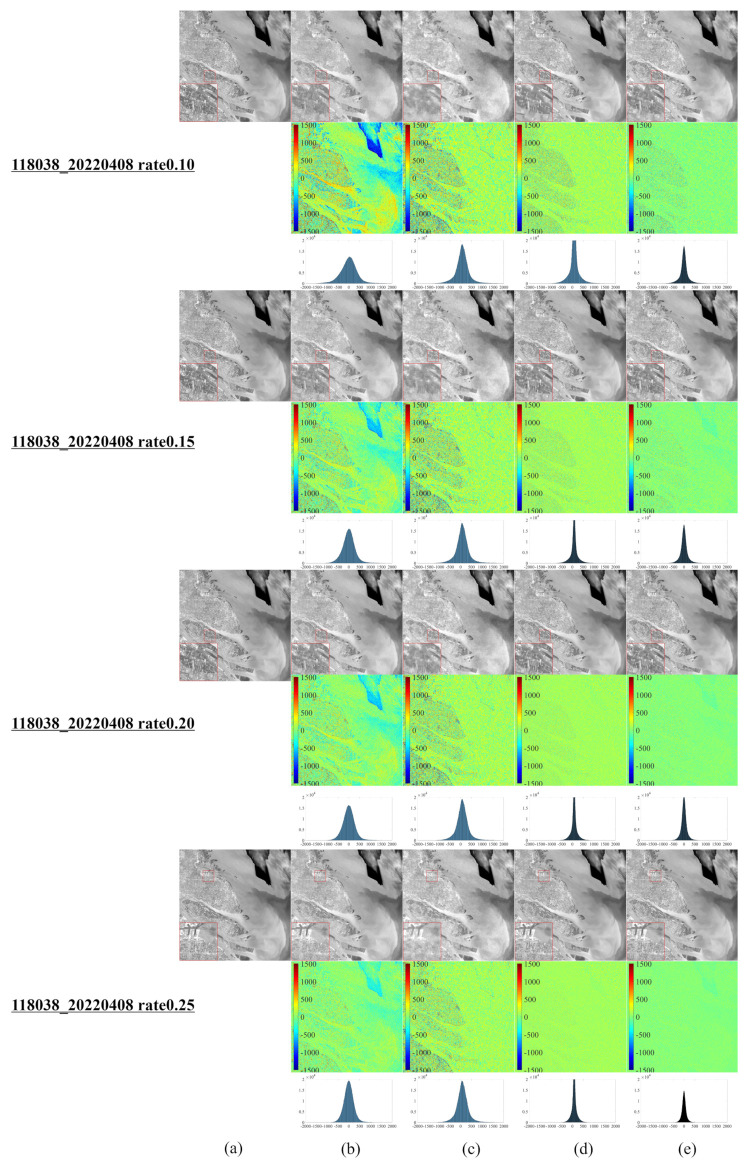
CS reconstruction result images of a Landsat 9 RSI in Shanghai, China, using various models ((**a**–**e**) similar to Figure 6 and the second left image in the second row of Figure 1).

**Figure 9:**
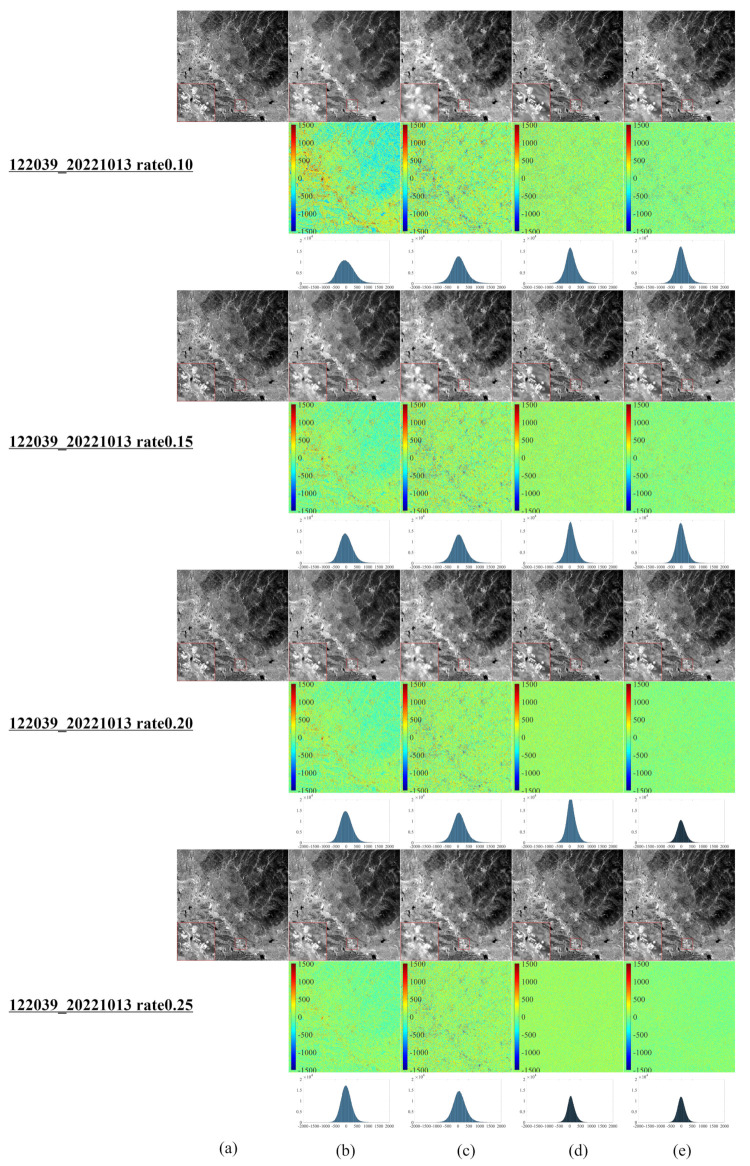
CS reconstruction result images of a Landsat 9 RSI in Huangshi, Hubei, China, using various models ((**a**–**e**) similar to Figure 6 and the fourth left image in the second row of Figure 1).

**Figure 10:**
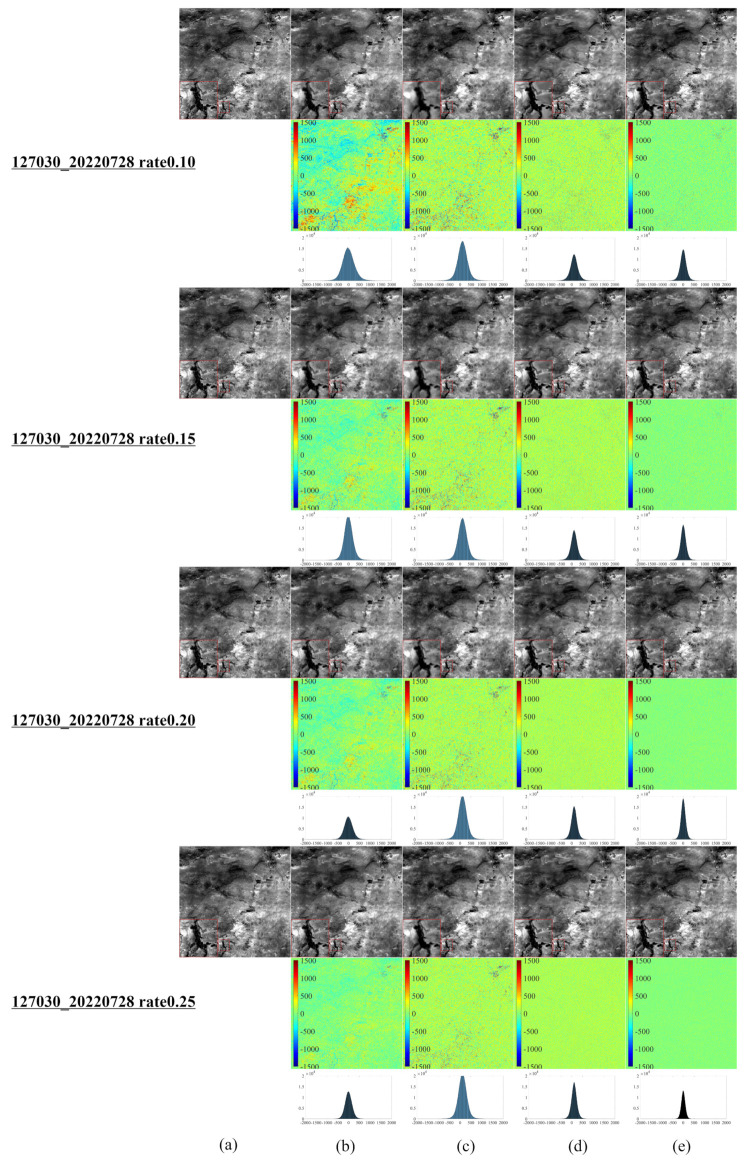
CS reconstruction result images of a Landsat 9 RSI in Erenhot, Inner Mongolia, China, using various models ((**a**–**e**) similar to Figure 6 and the last image in the second row of Figure 1).

To demonstrate the effectiveness of the newly developed method, extensive simulations were conducted using Landsat 8–9 RSIs. Since Landsat 8–9 RSIs (30-meter resolution) are too large, we adjusted the images by cropping, scaling, recombining, and synthesizing. After processing, the simulated Landsat 8–9 RSI had a 1000×1000 resolution used to evaluate the proposed CS model. Then, the CS model was applied to reconstruct the compressed sampling data and obtain the restored image, which was compared against the original image to assess the model. All simulations were conducted on an Intel (R) Core (TM) i9-10980XE CPU @ 3.00 GHz and with 128 GB RAM. The synthetic RSIs for reconstruction are illustrated in Figure 1:

Figure 1 illustrates a series of synthetic images obtained from the original Landsat 8–9 images, which were used to evaluate our algorithm after cutting and resizing. The above images were used in the newly developed CS model for reconstruction and comparative evaluation against other CS models, namely, NL-Laplace-CS, NL-SRF-CS [16], KCS-GSR [31], and NLDR-CS [32], and revealed the advantages and disadvantages of each model. The Landsat 8–9 RSIs were sampled using different frequency-domain sampling ratios (10%, 15%, 20%, and 25%) and were reconstructed using the above model. The corresponding reconstruction results are presented in Figure 2, Figure 3, Figure 4, Figure 5, Figure 6, Figure 7, Figure 8, Figure 9 and Figure 10.

Figure 2 reveals that the NL-Laplace-CS model performs better than NL-SRF-CS, KCS-GSR, and NLDR-CS considering reconstruction. From the first line (10%), the fourth line (15%), the seventh line (20%), and the tenth line (25%), it is evident that the images reconstructed by the NL-Laplace-CS model have no obvious edge blur, but the competitor method reconstructs images with a visible edge blur. The reconstructed image using the NL-SRF-CS model has information loss in surface features and heterogeneous regions of textures. The images reconstructed with the NL-Laplace-CS and KCS-GSR methods present fewer zigzag effects than the other models. The overall reconstruction result of the NLDR-CS model is better than the NL-Laplace-CS and KCS-GSR models but still slightly inferior to the NL-Laplace-CS model. The NL-Laplace-CS model has inherent noise suppression and, therefore, can suppress areas of high-frequency changes. However, from the different images in Figure 2e red magnified diagram at the lower left corner of each image), we observe that the NL-Laplace-CS model retains the most details.

Figure 3 illustrates the CS reconstruction results of the Landsat 8 RSI covering Selkirk, Manitoba, Canada, using the same model presented in Figure 2 Compared with the competitor models, the NL-SRF-CS and KCS-GSR models present large differences compared to the original images. Specifically, the images reconstructed with the NL-SRF-CS method have significant edge blurring and lost more details in areas with more edges. The images reconstructed with the KCS-GSR model have a sharp zigzag effect. Moreover, the results of images reconstructed by NLDR-CS show a greater data loss of surface features and edge areas than those reconstructed images using the NL-Laplace-CS model. The overall visual effect of the reconstructed image from the NL-Laplace-CS model has the highest fidelity relative to the original images.

Figure 4 depicts the CS reconstruction of a Landsat 8 RSI of Lake Flathead, Montana, USA, using the same model as in Figure 2 and Figure 3. The difference between the reconstructed image of the KCS-GSR model and the original image is the largest, followed by the NL-SRF-CS model and NLDR-CS model. However, the proposed NL-Laplace-CS model has the smallest difference from the original image. The reconstructed images of the NL-SRF-CS model have much edge blurring and loss of detail in the edge area, and the KCS-GSR reconstructed image also lost more details than the original image. The reconstructed image of the NLDR-CS model affords fine surface structure and edge information, but there is a minor blurring effect in the edge area. In the image reconstructed by the NL-Laplace-CS model, the partially magnified area (lower left red box diagram) reveals a good visual effect, while the details of edges and non-smooth areas are also well preserved.

Figure 5 illustrates the resulting images of CS reconstruction of Landsat 8 RSIs from Lincoln, Washington, USA, using the model of Figure 2. The difference images show that the reconstructed result images of the KCS-GSR model and the NLDR-CS model differ the most from the original image, and the proposed NL-Laplace-CS model differs the least. For KCS-GSR and NLDR-CS models, the reconstructed image’s local magnified area (red block diagram in the lower left corner) has a more obvious edge blur. Furthermore, the reconstructed images of the NL-SRF-CS model reveal that the reconstruction results are significantly serrated, and there is also a small fuzzy effect in the uneven areas. For the NL-Laplace-CS model, the reconstructed images afford a good visualization, the suppression of details is invisible, and the boundaries of each object region in the image are well preserved.

Table 2 summarizes the number of pixels within a range (±260) of around 0 for each image. The more pixels within a pixel value range around 0, the better the reconstruction effect. Table 2 highlights the fact that most pixels of the different numbers between the image reconstructed by the NL-Laplace-CS model and the original image are concentrated in the range $\left[-260,260\right]$. Moreover, Figure 2, Figure 3, Figure 4 and Figure 5 illustrate that the range $\left(0,260\right)$ of the pixel values is very small compared to 16-bit images (0–65535). Besides, Table 2 reveals that the percentage of pixel difference within the $\left[-260,260\right]$ range between the reconstructed image of the NL-Laplace-CS model and the original image is the largest (the percentage of the whole image), indicating that the fidelity of a Landsat 8 RSI reconstructed by the proposed NL-Laplace-CS model is the highest.

To further evaluate our model’s performance, we employ three quantitative image quality indicators (PQI): root mean square error (RMSE), peak signal-to-noise ratio (PSNR), and structural similarity (SSIM) [33]. A lower RMSE value, higher PSNR value, or higher SSIM value indicates a better model performance. Table 3 summarizes the PQI values in different areas for each model presented in Figure 2, Figure 3, Figure 4 and Figure 5. For the Antarctic region (Figure 2), the difference in the RMSE values between the competitor models is obvious, as the RMSE values of the NL-SRF-CS model are significantly larger, followed by the RMSE of the KCS-GSR model and NLDR-CS model. The NL-Laplace-CS model has the smallest RMSE among all models.

Moreover, the average error generated by the NL-Laplace-CS model in all geographic regions is the smallest among all models for all sampling ratios. For Selkirk in Manitoba, Canada (Figure 3), all models have similar PSNR values, but the PSNR values of NL-SRF-CS and KCS-GSR models are relatively smaller than the other models. Although the NLDR-CS model has high PSNR values, it changes relatively slowly with the sampling rate increasing. In any case, the PSNR of the NL-Laplace-CS model is the maximum. Furthermore, the PSNR results (see Table 3) indicate that the NL-Laplace-CS model performs well, and its SSIM value is greater than the competitor models. Although the SSIM value increases with the image sampling rate, the rate at which the new model increases is much larger than the other models. These PQI results indicate that the newly developed NL-Laplace-CS model has lower RMSE values, higher PSNR and SSIM values, better visual effects, and edge preservation.

The second group of satellite images used in our simulations involves the Landsat 9 L2SP product in the red band (30-meter) with a wavelength range of 640–670 nm. Similar to the Landsat 8 synthetic simulation images, the Landsat 9 image was trimmed and scaled to obtain the simulation Landsat 9 data presented in the second row in Figure 1. Finally, we evaluated that dataset with the NL-SRF-CS, KCS-GSR, NLDR-CS, and NL-Laplace-CS models and analyze the performance of each algorithm through the simulation results.

Figure 6 illustrates the CS reconstruction images of the Landsat 9 RSIs from Lake Abitibi, Ontario, Canada, using the model presented in Figure 2. In the algorithm simulation experiment in this region, various CS reconstruction models can reconstruct the resulting images well. However, there are still some differences, as Figure 6 highlights the fact that the NL-SRF-CS model has a good reconstruction effect in the relatively smooth area, but the reconstruction effect in the edge area and the detailed area is unsatisfactory. The reconstructed resulting images of the KCS-GSR model are quite different from the original images; namely, there is a certain gap between the overall reconstruction results of the KCS-GSR model and those of the NLDR-CS model and NL-Laplace-CS model. For NL-Laplace-CS and NLDR-CS models, the reconstructed image’s local magnified area (red block diagram in the lower left corner) has more obvious detail preservation. At the same time, the reconstruction results of the proposed NL-Laplace-CS model have the least difference from the original images.

Figure 7 depicts the resulting images of the CS reconstruction of the Landsat 9 RSI of Yeosu, South Korea, using the model presented in Figure 2. For this area, the reconstructed images of all CS reconstruction models present unsatisfactory results, mainly due to the geographic details in this region. Figure 7 reveals that the KCS-GSR model’s reconstructed images differ the most from the original ones, followed by the NL-SRF-CS model. The NLDR-CS model shows better reconstruction results but is still imperfect in the marginal and detailed areas. The reconstruction results of the proposed NL-Laplace-CS model present minor differences from the original images and thus affords the best visual effect.

Figure 8, Figure 9 and Figure 10 demonstrate each competitor algorithm’s advantages and disadvantages based on each image’s corresponding reconstruction result. However, due to paper length limitations, we neglect a detailed analysis. Additionally, Table 4 summarizes the number of pixels within a range (±260) of around 0 for different Landsat 9 images. The more pixel values within the range around 0, the better the reconstruction effect. Table 4 reveals that most pixels of the NL-Laplace-CS model-based reconstructed images are concentrated within the range $\left[-260,260\right]$, as illustrated in Figure 6, Figure 7, Figure 8, Figure 9 and Figure 10. For 16-bit images (0 to 65,535), the range of (0 to 260) is very small. Table 4 highlights the fact that the NL-Laplace-CS model has the largest percentage of pixels (percentage of the whole image) in a range of around 0, indicating that its performance is best among the models evaluated.

To further evaluate the proposed model’s performance, we calculate the RMSE, PSNR, and SSIM values on the CS reconstructed Landsat 9 images. Table 5 summarizes the information presented in Figure 6, Figure 7, Figure 8, Figure 9 and Figure 10 and highlights the fact that the NL-Laplace-CS model has the lowest RMSE value and the highest PSNR and SSIM values. The resulting images used for the CS reconstruction based on the NL-Laplace-CS model had the best visual effect, namely, the most preserved edge information and image details.

## 7. Discussion

The NL-Laplace-CS regularization method relies on the appropriate adjustment of the data fidelity and weight parameters in the regularization terms. With the optimal choice of the weight parameters, this method yields the best reconstruction results. The experiments use the Landsat 8–9 RSI for CS simulation reconstruction, and we compared the reconstruction results obtained from the Landsat 8–9 RSI simulation with the NL-Laplace-CS model against the NL-SRF-CS, KCS-GSR, and NLDR-CS image reconstruction models. We also compared these reconstruction results with the original Landsat 8–9 RSI. The results highlighted that the new NL-Laplace-CS had lower RMSE values, higher PSNR and SSIM values, and better visual effects than the competitor models. Errors mostly occur between geological structures and land cover classes in the transition zone. The resulting images of the CS reconstruction of the newly developed NL-Laplace-CS model reveal that, even in such a transition zone, the reconstructed pixel values still have a high fidelity relative to the original Landsat 8–9 RSIs. This is important because most remote sensing image applications require high fidelity in the transition zone to clarify the geological surface features with complex geographical compositions.

## 8. Conclusions

This work developed a Landsat 8–9 RSI CS reconstruction model based on a non-local framework combined with low-rank regularization approximation of non-convex Laplace functions, which was solved using the ADMM algorithm. The proposed NL-Laplace-CS model was challenged against advanced CS image reconstruction models such as NL-SRF-CS, KCS-GSR, and NLDR-CS in a simulation reconstruction for Landsat 8–9 RSIs. The simulation results revealed that the proposed NL-Laplace-CS model effectively utilized similar image patches of sparse groups and established a low-rank regularized approximate minimization model of non-convex Laplace functions, suggesting a very effective method for CS reconstruction of Landsat 8–9 RSIs. Specifically, the proposed NL-Laplace-CS model has lower RMSE values, higher PSNR and SSIM values, and outperforms state-of-the-art CS image reconstruction models such as NL-SRF-CS, KCS-GSR, and NLDR-CS.

## Figures and Tables

**Table 1:** The images and regions used for the CS reconstruction algorithm of remote sensing image.

Image Locations and Date			
**Remote Sensing Image Type**	**Locations**	**WRS2** **(Worldwide Reference System 2)**	**Date**
Landsat 8	Antarctica	128111	22/02/2018
	Selkirk, Manitoba, Canada	031025	07/10/2022
	Lake Flathead, Montana, USA	041027	29/09/2017
	Lincoln, Washington, USA	044027	15/10/2015
Landsat 9	Lake Abitibi, Ontario, Canada	019026	11/10/2022
	Yeosu, Republic of Korea	115036	12/10/2022
	Shanghai, China	118038	08/04/2022
	Huangshi, Hubei, China	122039	13/10/2022
	Erenhot, Inner Mongolia, China	127030	28/07/2022

**Table 2:** Percentage of the number of pixels (in a small range $\left[-260,260\right]$ of around 0) of the difference images between the original Landsat 8 RSI and the reconstructed image using different CS models.

Landsat 8 Remote Sensing Image	Range of Pixel Value	Rate	Percentage of Pixels Occupied							
			NL-SRF-CS		KCS-GSR		NLDR-CS		NL-Laplace-CS	
			Inside	Outside	Inside	Outside	Inside	Outside	Inside	Outside
Antarctica 128111_20180222	[−260,260]	0.10	31.7318	68.2682	51.8176	48.3557	70.9197	29.0803	86.5486	13.4514
	[−260,260]	0.15	40.9783	59.0217	55.6457	44.5336	77.8590	22.1410	94.2010	5.7990
	[−260,260]	0.20	44.6930	55.3070	58.5099	41.6791	82.7760	17.2240	97.9707	2.0293
	[−260,260]	0.25	54.7053	45.2947	61.4826	38.7127	86.6517	13.3483	99.6080	0.3920
Selkirk, Manitoba, Canada 031025_20221007	[−260,260]	0.10	30.7078	69.2922	38.9215	61.2166	60.4930	39.5070	66.7224	33.2776
	[−260,260]	0.15	36.9558	63.0442	42.0941	58.0497	68.5670	31.4330	74.7742	25.2258
	[−260,260]	0.20	39.8745	60.1255	45.0647	55.0905	75.5086	24.4914	82.8788	17.1212
	[−260,260]	0.25	46.7389	53.2611	47.8097	52.3493	81.5440	18.4560	90.4872	9.5128
Flathead Lake, Montana, USA, 041027_20170929	[−260,260]	0.10	77.4191	22.5809	78.4541	21.7012	84.8593	15.1407	93.9916	6.0084
	[−260,260]	0.15	87.4137	12.5863	80.5140	19.6338	88.0287	11.9713	96.8053	3.1947
	[−260,260]	0.20	88.3001	11.6999	82.6323	17.5099	90.4024	9.5976	98.8148	1.1852
	[−260,260]	0.25	93.2202	6.7798	83.7051	16.4393	92.2663	7.7337	99.6793	0.3207
Lincoln, Washington, USA 044027_2015288	[−260,260]	0.10	86.4147	13.5853	81.8592	18.3014	85.6378	14.3622	96.8269	3.1731
	[−260,260]	0.15	94.4427	5.5573	87.7942	12.3480	89.0729	10.9271	98.6488	1.3512
	[−260,260]	0.20	95.1954	4.8046	86.1578	13.9878	91.6887	8.3113	99.6090	0.3910
	[−260,260]	0.25	97.9079	2.0921	87.7942	12.3480	93.7166	6.2834	99.9129	0.0871

**Table 3:** RMSE, PSNR, and SSIM values for different models in the Landsat 8 remote sensing image (RSI) study shown in Figure 2, Figure 3, Figure 4 and Figure 5.

Landsat 8 Remote-Sensing Images	PQIs		NL-SRF-CS	KCS-GSR	NLDR-CS	NL-Laplace-CS
Antarctica 128111_20180222	Rate0.10	RMSE	898.253	480.6734	203.4486	**195.7073**
		PSNR	37.2615	43.2624	50.0925	**50.4973**
		SSIM	0.9415	0.97808	0.9935	**0.9942**
	Rate0.15	RMSE	641.1155	412.0085	153.7279	**130.5971**
		PSNR	40.1907	44.8054	52.5266	**54.0107**
		SSIM	0.9598	0.9828	0.9959	**0.9971**
	Rate0.20	RMSE	539.2623	373.2582	124.7959	**93.5328**
		PSNR	41.6935	45.8467	54.3376	**56.9102**
		SSIM	0.9664	0.98561	0.9971	**0.9984**
	Rate0.25	RMSE	401.2769	326.7187	104.8497	**68.3434**
		PSNR	44.2606	47.3134	55.8503	**59.6355**
		SSIM	0.978	0.98882	0.9979	**0.9991**
Selkirk, Manitoba, Canada, 031025_20221007	Rate0.10	RMSE	833.0314	699.6305	360.0842	**307.8145**
		PSNR	37.9162	39.5301	45.1335	**46.5637**
		SSIM	0.924	0.93774	0.9777	**0.9834**
	Rate0.15	RMSE	639.0923	620.12	276.0758	**242.2749**
		PSNR	40.2182	40.6163	47.441	**48.6433**
		SSIM	0.9447	0.9476	0.9859	**0.9893**
	Rate0.20	RMSE	555.8848	553.2539	220.699	**190.908**
		PSNR	41.4298	41.6514	49.3856	**50.713**
		SSIM	0.9543	0.95653	0.9906	**0.9931**
	Rate0.25	RMSE	446.627	501.8709	179.8765	**149.425**
		PSNR	43.3306	42.5392	51.1621	**52.841**
		SSIM	0.9948	0.9633	0.9935	**0.9957**
Flathead Lake, Montana, USA 041027_20170929	Rate0.10	RMSE	251.2652	243.7577	177.0215	**130.6265**
		PSNR	48.3268	49.3549	51.3011	**54.0088**
		SSIM	0.9915	0.992	0.9938	**0.9964**
	Rate0.15	RMSE	190.7938	226.0831	152.4969	**109.2699**
		PSNR	50.7182	50.1743	52.5964	**55.5595**
		SSIM	0.9939	0.99292	0.9951	**0.9974**
	Rate0.20	RMSE	178.4575	206.608	134.7184	**88.429**
		PSNR	51.2988	51.1945	53.6731	**57.3976**
		SSIM	0.9945	0.99396	0.996	**0.9982**
	Rate0.25	RMSE	147.1679	198.9782	120.6403	**72.7902**
		PSNR	52.9732	51.6346	54.6318	**59.088**
		SSIM	0.9959	0.99447	0.9967	**0.9988**
Lincoln, Washington, USA, 044027_2015288	Rate0.10	RMSE	170.9491	197.1138	163.8181	**110.3724**
		PSNR	51.6721	51.7843	51.9744	**55.4723**
		SSIM	0.9953	0.99505	0.995	**0.9974**
	Rate0.15	RMSE	129.7697	184.5744	142.788	**92.3254**
		PSNR	54.066	52.6122	53.1678	**57.023**
		SSIM	0.9967	0.99566	0.9959	**57.023**
	Rate0.20	RMSE	125.595	174.4205	126.319	**75.308**
		PSNR	54.35	53.3213	54.2322	**58.7926**
		SSIM	0.997	0.99616	0.9966	**0.9987**
	Rate0.25	RMSE	105.3094	166.1922	113.3335	**62.0297**
		PSNR	55.8801	53.9867	55.1744	**60.4775**
		SSIM	0.9977	0.9966	0.9972	**0.9991**

**Table 4:** Number of pixels in a small range around 0 (in percentage) of the difference images between the original Landsat 9 RSI and the reconstructed image using different CS models.

Landsat 9 Remote Sensing Image	Range of Pixel Value	Rate	Percentage of Pixels Occupied							
			NL-SRF-CS		KCS-GSR		NLDR-CS		NL-Laplace-CS	
			Inside	Outside	Inside	Outside	Inside	Outside	Inside	Outside
Lake Abitibi, Ontario, Canada 019026_20221011	[−260,260]	0.10	86.1794	13.8206	81.8307	18.3343	86.3252	13.6748	95.4589	4.5411
	[−260,260]	0.15	93.3402	6.6598	83.7116	16.4469	88.6563	11.3437	97.7012	2.2988
	[−260,260]	0.20	94.2158	5.7842	85.2966	14.8484	90.6463	9.3537	99.1153	0.8847
	[−260,260]	0.25	97.1915	2.8085	86.5466	13.5959	92.3951	7.6049	99.7508	0.2492
Yeosu, Korea 115036_20221012	[−260,260]	0.10	56.7899	43.2101	66.3285	33.8273	80.7143	19.2857	82.9278	17.0722
	[−260,260]	0.15	70.6925	29.3075	68.2372	31.9191	84.9258	15.0742	87.3849	12.6151
	[−260,260]	0.20	72.4175	27.5825	69.6345	30.5274	88.2334	11.7666	92.1732	7.8268
	[−260,260]	0.25	79.8082	20.1918	70.9790	29.1828	91.0656	8.9344	96.0945	3.9055
Shanghai, China 118038_20220408	[−260,260]	0.10	53.9526	46.0474	65.2240	34.9397	79.8067	20.1933	83.3926	16.6074
	[−260,260]	0.15	65.8213	34.1787	66.5302	33.6376	84.3560	15.6440	87.1873	12.8127
	[−260,260]	0.20	67.4619	32.5381	67.9013	32.2696	88.0579	11.9421	92.1889	7.8111
	[−260,260]	0.25	75.5923	24.4077	68.7766	31.3947	91.1311	8.8689	96.2243	3.7757
Huangshi, Hubei, China 122039_20221013	[−260,260]	0.10	49.5321	50.4679	55.5235	44.6434	65.5933	34.4067	68.3807	31.6193
	[−260,260]	0.15	60.2258	39.7742	57.7512	42.4289	71.2787	28.7213	73.0715	26.9285
	[−260,260]	0.20	63.5580	36.4420	59.7541	40.4251	76.4113	23.5887	78.8740	21.1260
	[−260,260]	0.25	70.9719	29.0281	61.6484	38.5290	81.1467	18.8533	84.3839	15.6161
Erenhot, Inner Mongolia, China 127030_20220728	[−260,260]	0.10	64.5972	35.4028	67.3637	32.8217	75.7473	24.2527	88.7506	11.2494
	[−260,260]	0.15	78.5930	21.4070	69.7726	30.4136	79.7862	20.2138	92.6547	7.3453
	[−260,260]	0.20	79.6510	20.3490	71.6206	28.5706	83.0796	16.9204	95.9827	4.0173
	[−260,260]	0.25	87.1182	12.8818	73.0742	27.1169	85.7812	14.2188	98.2061	1.7939

**Table 5:** RMSE, PSNR, and SSIM values of the different models in the Landsat 9 RSI study, as illustrated in Figure 6, Figure 7, Figure 8, Figure 9 and Figure 10.

Landsat 9 Sensing Image	PQIs		NL-SRF-CS	KCS-GSR	NLDR-CS	NL-Laplace-CS
Lake Abitibi, Ontario, Canada 019026_20221011	Rate0.10	RMSE	178.6558	202.9490	165.8346	**122.6032**
		PSNR	51.2891	51.3389	51.8681	**54.5594**
		SSIM	0.9946	0.9942	0.9945	**0.9967**
	Rate0.15	RMSE	139.6148	191.3931	148.7677	**104.8770**
		PSNR	53.4308	52.0435	52.8114	**55.9159**
		SSIM	0.9961	0.9948	0.9953	**0.9975**
	Rate0.20	RMSE	133.2829	181.8600	135.4003	**88.0589**
		PSNR	53.8340	52.6628	53.6292	**57.4340**
		SSIM	0.9965	0.9954	0.9960	**0.9982**
	Rate0.25	RMSE	112.5607	174.6843	124.1275	**74.0333**
		PSNR	55.3017	53.1794	54.3842	**58.9409**
		SSIM	0.9973	0.9958	0.9965	**0.9987**
Yeosu, Korea, 115036_20221012	Rate0.10	RMSE	357.9920	352.5152	234.8635	**211.7692**
		PSNR	45.2520	45.7370	48.8453	**49.8122**
		SSIM	0.9809	0.9805	0.9885	**0.9907**
	Rate0.15	RMSE	277.7287	325.7535	191.6372	**175.6439**
		PSNR	47.4571	46.4924	50.6120	**51.4368**
		SSIM	0.9860	0.9826	0.9920	**0.9935**
	Rate0.20	RMSE	255.6346	308.1822	160.4600	**140.0482**
		PSNR	48.1771	47.0331	52.1543	**53.4039**
		SSIM	0.9880	0.9842	0.9943	**0.9958**
	Rate0.25	RMSE	212.1976	290.5371	136.3814	**111.8590**
		PSNR	49.7947	47.6095	53.5665	**55.3560**
		SSIM	0.9911	0.9857	0.9958	**0.9972**
Shanghai, China, 118038_20220408	Rate0.10	RMSE	421.4962	375.1064	235.0718	**213.3513**
		PSNR	43.8336	45.2743	48.8376	**49.7476**
		SSIM	0.9767	0.9787	0.9886	**0.9908**
	Rate0.15	RMSE	317.1140	353.8817	185.3175	**178.0600**
		PSNR	46.3052	45.8320	50.9033	**51.3181**
		SSIM	0.9832	0.9805	0.9926	**0.9935**
	Rate0.20	RMSE	290.2904	327.6273	150.3745	**137.9961**
		PSNR	47.0728	46.5834	52.7181	**53.5321**
		SSIM	0.9854	0.9828	0.9950	**0.9960**
	Rate0.25	RMSE	236.4489	316.6613	123.8975	**108.0126**
		PSNR	48.8547	46.9231	54.4004	**55.6600**
		SSIM	0.9894	0.9840	0.9966	**0.9975**
Huangshi, Hubei, China 122039_20221013	Rate0.10	RMSE	396.5857	388.7419	292.8481	**277.0668**
		PSNR	44.3627	44.8508	46.9288	**47.4778**
		SSIM	0.9755	0.9751	0.9820	**0.9840**
	Rate0.15	RMSE	318.2901	362.8340	249.2427	**243.6536**
		PSNR	46.2730	45.5082	48.3292	**48.5940**
		SSIM	0.9812	0.9775	0.9863	**0.9874**
	Rate0.20	RMSE	291.5050	341.4579	215.0280	**209.8189**
		PSNR	47.0365	46.0784	49.6117	**49.8926**
		SSIM	0.9839	0.9797	0.9895	**0.9904**
	Rate0.25	RMSE	247.1052	324.2598	185.9369	**180.8646**
		PSNR	48.4718	46.5733	50.8743	**51.1824**
		SSIM	0.9877	0.9815	0.9920	**0.9928**
Erenhot, Inner Mongolia, China 127030_20220728	Rate0.10	RMSE	303.5128	291.5269	202.6761	**167.4437**
		PSNR	46.6859	48.0452	50.1256	**51.8521**
		SSIM	0.9879	0.9888	0.9919	**0.9942**
	Rate0.15	RMSE	222.9116	271.3286	173.9108	**141.6788**
		PSNR	49.3668	48.8500	51.4551	**53.3034**
		SSIM	0.9915	0.9900	0.9936	**0.9957**
	Rate0.20	RMSE	210.7621	255.6723	152.7735	**118.1105**
		PSNR	49.8536	49.5255	52.5806	**54.8837**
		SSIM	0.9923	0.9911	0.9949	**0.9969**
	Rate0.25	RMSE	171.3273	244.1732	136.6703	**98.9114**
		PSNR	51.6529	50.0810	53.5481	**56.4245**
		SSIM	0.9944	0.9919	0.9958	**0.9978**

## Data Availability

The data presented in this study are available on request from the corresponding author.
